# Decision modelling of non-pharmacological interventions for individuals with dementia: a systematic review of methodologies

**DOI:** 10.1186/s13561-018-0192-8

**Published:** 2018-03-26

**Authors:** Elizaveta Sopina, Jan Sørensen

**Affiliations:** 1 Centre of Health Economics (COHERE), Department of Public Health, University of Southern Denmark, J.B. Winsløws Vej 9, 5000 Odense, Denmark; 2 Healthcare Outcomes Research Centre, Royal College of Surgeons in Ireland, Dublin, Ireland

**Keywords:** Dementia, Economic evaluation, Decision modelling, Non-pharmacological interventions

## Abstract

**Objectives:**

The main objective of this study is to conduct a systematic review to identify and discuss methodological issues surrounding decision modelling for economic evaluation of non-pharmacological interventions (NPIs) in dementia.

**Methods:**

A systematic search was conducted for publications using decision modelling to investigate the cost-effectiveness of NPIs for individuals with dementia. Search was limited to studies in English. Studies were excluded if they evaluated interventions aimed only at caregivers of patients with dementia, or if they only included economic evaluation alongside an RCT without additional modelling.

**Results:**

Two primary, five secondary and three tertiary prevention intervention studies were identified and reviewed. Five studies utilised Markov models, with others using discrete event, regression-based simulation, and decision tree approaches. A number of challenging methodological issues were identified, including the use of MMSE-score as the main outcome measure, limited number of strategies compared, restricted time horizons, and limited or dated data on dementia onset, progression and mortality. Only one of the three tertiary prevention studies explicitly considered the effectiveness of pharmacological therapies alongside their intervention.

**Conclusions:**

Economic evaluations of NPIs in dementia should utilise purposefully-developed decision models, and avoid models for evaluation of pharmaceuticals. Broader outcome measures could be a way to capture the wide impact of NPIs for dementia in future decision models. It is also important to account for the effects of pharmacological therapies alongside the NPIs in economic evaluations. Access to more localised and up-to-date data on dementia onset, progression and mortality is a priority for accurate prediction.

**Electronic supplementary material:**

The online version of this article (10.1186/s13561-018-0192-8) contains supplementary material, which is available to authorized users.

## Background

Dementia is a common syndrome, mostly affecting the elderly and is characterised by progressive loss of memory and cognitive function [1]. Estimates suggest that the prevalence of dementia is between 5.9 and 9.4% amongst people aged 65 and over [2, 3]. Both the number of people with dementia and costs of treatment have been rising rapidly in the past two and a half decades [4]. Given the impending ageing population and limited health care resources, reliable and valid cost-effectiveness analyses of interventions targeted at people with dementia is an important task for both researchers and policy makers [5].

Most of interventions available for people with dementia are of pharmacological nature, aiming to either relieve symptomatic aspects of the condition, or delay the process of cognitive deterioration [6]. Although these have had a positive impact, the scope and effectiveness of these drugs have been questioned [4, 7]. In particular, available pharmaceuticals appear to have little or no effect on behavioural and functional outcomes [8]. The high cost and limited success in developing new pharmaceuticals has led to suggestions that a different, broader approach to dealing with dementia is required [7].

Such an approach can include delaying onset by controlling modifiable lifestyle factors, such as smoking, obesity and exercise (primary prevention), timely identification of the disease (secondary prevention) and post-diagnostic interventions aimed at improving quality of life and delaying progression of dementia (tertiary prevention) [4, 9, 10]. A broader approach to dealing with dementia is given increasing importance and a growing number of studies investigating novel, non-pharmacological approaches to treating, managing and supporting people with dementia and their carers have been emerging [11]. In fact, the number of studies investigating the effectiveness of non-pharmacological interventions (NPIs) has been growing fast in the last 15 years [10]. These interventions target a wide spectrum of dementia symptoms: not only cognitive decline, as most pharmaceuticals, but also psychological, behavioural aspects, as well as functional abilities/activities of daily living, among others [10, 12–15].

While some evidence for cost-effectiveness of NPIs exists [16], the majority of economic evaluations of dementia interventions focus on pharmacological therapies. Contrasted with the number of NPIs available, a wide gap in economic evaluation of such interventions is evident: in their review of decision analytic models for Alzheimer’s disease (AD; the most prevalent type of dementia), Cohen and Neumann [17] found that many of the existing models for economic evaluation of pharmacological AD interventions are not suited for assessment of NPIs. In fact, all economic models aimed at evaluating interventions for dementia identified in a systematic review by Green, Shearer, Ritchie and Zajicek et al. [18] were created for the purpose of evaluating cost-effectiveness of a pharmaceutical.

Further, application of pharmacological models to NPIs is problematic, given the differences in aims, scope and assumptions behind the two types of interventions. While the main concern of pharmacological interventions is slowing the progression of dementia, NPIs often have a much broader scope, thus requiring an outcome measure with a broader scope than just cognition. NPIs also consider the interaction between the patient, their caregiver and their environment, including the medical and support systems in place – something that is often disregarded in pharmacological evaluations [10, 13, 15]. The time horizon of NPIs, and, in particular, those with a preventative or screening focus, is also likely to be different to pharmacological therapies. It is therefore reasonable to expect that methodologies for evaluation of NPIs should be different from those used for evaluating pharmacological interventions.

A recent review has demonstrated that pharmacological treatments are significantly costlier than NPIs, thus posing a significantly higher economic burden on healthcare budgets [4]. However, the authors did not go as far as to compare cost-effectiveness of pharmacological interventions with NPIs, partially due to the short-term nature of trials available, as well as small numbers of people participating in those. A potential solution to this is extrapolation of data and characterising uncertainty through decision modelling.

Given the growing impact of dementia on health outcomes and services, and the continuing development of new NPIs, it is important to foster a strong understanding of methodologies available for economic decision modelling in dementia. A number of existing reviews have investigated decision modelling in dementia [17–19], but none, to the best of our knowledge, have focussed solely on NPIs.

This paper is aimed at researchers and policy makers working on economic evaluation of NPIs for dementia. In this paper we set out to provide a detailed overview of current methods available for decision modelling of NPIs for people with dementia, identify and discuss methodological issues surrounding decision modelling for economic evaluation of non-pharmacological interventions (NPIs) in dementia, in particular with respect to model structure, outcome measures and data inputs.

## Methods

The systematic review followed the methodology proposed by the Cochrane Handbook for Systematic Reviews of Interventions and was reported in accordance with Preferred Reporting Items for Systematic Reviews and Meta-Analyses (PRISMA) [20, 21].

### Search sources and strategy

A literature search was conducted on the 7th July 2017 on PubMed, Scopus, Science Direct, Cochrane, NHS EED, Embase, EconLit and Psychinfo databases. Detailed search strategy for each database is presented in Additional file 1. The search was limited to studies published in English. The search did not include studies published prior to 2000, for a number of reasons: there were few NPIs for dementia developed before the year 2000 [10], studies before 2000 were covered by broader reviews and do not reveal many decision models on NPIs [17–19], and modelling techniques used prior to 2000 are likely to have been significantly improved and perfected [22].

Study eligibility was established on initial screening of title and abstract. Studies passing initial screening were subject to full text review against inclusion and exclusion criteria, outlined in Table 1. Studies were included if they modelled the onset and/or progression of dementia, and evaluated the cost-effectiveness of a NPI aimed at people at risk of or with dementia. All study designs were considered for review. Studies were excluded if they evaluated interventions aimed only at caregivers of patients with dementia, or if they only included economic evaluation alongside an RCT without additional modelling.

**Table 1:** Inclusion and exclusion criteria

**Inclusion Criteria**	**Exclusion Criteria**
- Original decision model - Study models onset and/or progression of dementia - Published in or after 2000 - Assesses health-economic impact of intervention through cost-effectiveness, -benefit, -utility or -minimisation analysis - Assesses a non-pharmacological intervention targeting dementia	- Pharmacological intervention only - Intervention targeting solely caregivers of patients with dementia

### Review process

The nature of the intervention should, at least in part, dictate the structure of the model, and, therefore the necessary inputs such as transition probabilities, utilities, costs etc. [22]. Therefore, the analysis of studies selected for this review is focused on three categories of NPIs: primary, secondary and tertiary prevention, as defined previously. Primary prevention includes interventions aiming to preventative or delay onset of dementia, secondary prevention interventions include screening and early identification initiatives, whilst interventions in the tertiary prevention category focus on aiding the symptoms and/or slowing the progression of the disease after a diagnosis has been made. Key features of the included studies were summarised, with specific focus on model structure, outcome measures and characterisation of disease onset and progression. These were selected to reflect the most significant aspects of modelling dementia-related interventions [18, 23], with particular relevance to evaluation of NPIs.

Methodological approaches were described with a view to identify common challenges and possible improvements for future model-based economic evaluations of NPIs for dementia. This was done by examining the studies against two widely-used best-practice guidelines for decision modelling in economic evaluation [24, 25]. Data used in reviewed models were assessed for the relevance in terms of date, setting, sample size and method of collection. In cases where studies utilised secondary/published data, we reviewed the data at the cited source.

## Results

**Fig. 1:**
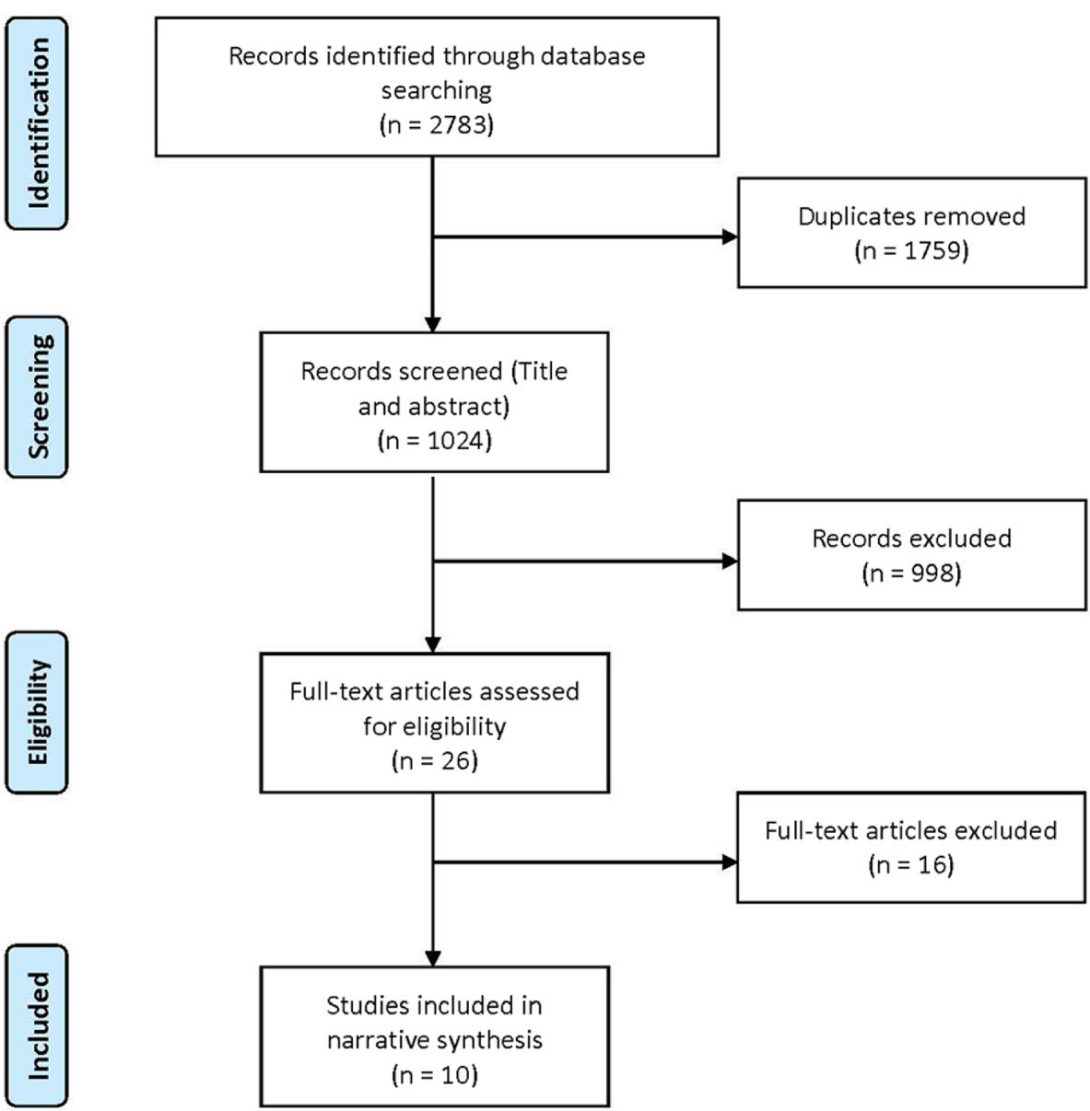
Flow chart for selection of studies included in the systematic review

After duplicates were removed, 1024 studies were identified, of which 998 were excluded after the initial screening (Fig. 1). The main reasons for exclusion were: pharmacological nature of the intervention; models not simulating dementia onset or progression, or interventions that solely targeted caregivers of AD or dementia patients. We also excluded systematic reviews and editorials, as well as studies not written in English, and those that were not AD- or dementia-specific in their focus. Studies utilising the same model as an already included study were also excluded from this review.

A total of ten studies were included in the review. An overview of the selected studies is presented in Table 2 and each study is briefly summarised in Additional file 2.

**Table 2:** Summary of included studies

	Model (reference)	Intervention and setting	Main outcome(s)	Modelling approach/ framework, time horizon and cycle	Data sources			
					Disease onset, progression and mortality data	Intervention effectiveness	Utilities/outcomes measure	Costs
Primary prevention	Zhang, Kivipelto [26]	Hypothetical intervention reducing risk of AD onset in Sweden	QALYs	Purpose-built Markov model with 3 health states. 20-year time horizon, 1 year cycles	CAIDE population based study on risk factors of 1409 individuals [44]	Hypothetical intervention	Swedish studies (EQ5D) [41, 57]	Swedish National Board of Health and Welfare
	Tsiachristas & Smith [32]	Preventative treatment with B-vitamin supplement for people aged 60 and over with elevated levels of tHcy in the UK	Life-Years; QALYs	Stochastic probabilistic decision tree; lifetime horizon.	Disease progression not modelled. Disease onset based on prevalence data; mortality from life tables.	Effectiveness of intervention based on a systematic review in lieu of randomised controlled trials [37]	General population EQ5D survey [36]	Taken from a UK study [58]
Secondary prevention	McMahon [28]	Functional neuroimaging vs. standard work-up of patients for AD diagnosis at specialised AD clinics in the US	QALYs	Markov model based on a previously published study [40]; 6-week cycles, 18-month time horizon.	Progression within AD and AD mortality from CERAD study [40]. Non-AD mortality from CDC.	Screening effectiveness from US-based study [59]	Utility weights obtained from the Neumann et al. [40]	Primary data from hospital databases; existing literature
	Silverman, Gambhir [31]	PET vs. standard diagnostic methods in clinical diagnosis of AD in the US	Number of accurate diagnoses	Purpose-built decision-tree, unspecified time horizon	Adapted from a wide range of published data	Results of PET screening reported in the study	Not used - CEA	Defined by Medicare reimbursement rates
	Weimer and Sager [30]	Early detection and treatment of AD patients in a US (Wisconsin) setting. Two treatments considered.	MMSE score change	Monte Carlo model. Lifetime horizon, 1 year cycles	Adapted from a range of published data and estimates. Data from CVD risk study on 5000 people was used to estimate hazard ratio for death.	A range of published data and estimates	Adapted from [40]	A range of published data and estimates
	Dixon, Ferdinand [35]	One-off screen of 75 year olds in England and Wales	Number of additional diagnoses	Static decision model with lifetime time horizon	Not provided	Results of screening based on MMSE (assumed 89% sensitivity, 95.5% specificity)	Not used – CBA	A range of published data and estimates
	Saito, Nakamoto [27]	Community based dementia screening in a US setting	Dementia diagnosis through MMSE	Purpose-built Markov model with 6-state 10-year time horizon, 1 year cycles	Adapted from [46, 48] which investigated 61 and 1145 patients, respectively	Results of screening program reported in study	Not used - CEA	Adapted from a Canadian study [60]
Tertiary prevention	McDonnell, Redekop [33]	A hypothetical intervention which slows cognitive decline in AD patients in the Netherlands	MMSE score change, care setting, mortality	Two regression-based simulation models – one modelling MMSE score, another- care setting and mortality. 10-year time horizon, 6 month cycles	Calculated from a Dutch study [38] with 7528 participants.	Hypothetical intervention	Not used – CEA	From Dutch national data, agencies/ ministries
	Martikainen, Valtonen [29]	Cognitive-behavioural family intervention to delay admission to nursing home in Finland	Time to nursing home admission	Markov model. Adapted from [40]. Model has 4 states, 5-year time horizon, 1 year cycles	Adapted from the original US-based model (with minor adjustments) – based on longitudinal study with 1145 patients [40]	Based on a US study of 206 subjects [61]	From the original US-based model	From national datasets; some resource utilisation based on expert panel
	Mirsaeedi-Farahani, Halpern [34]	Deep-brain stimulation therapy for slowing memory loss in AD patients compared to standard treatment	QALYs	Purpose-built Markov model with 5 states, 5-year horizon, 1 year cycles	Adapted from Neumann et al. [46] and Spackman et al. [47]	Actual success rate of deep brain stimulation is unknown, so was varied from 0 to 100%	A range of published data	Costs obtained from [62]

### Approach to modelling and model structure

Structure of the model should be transparent and justified given the aim, scope and perspective of the model [25]. It should also be possible to reproduce the model from the technical information provided to the reader [24].

Of the ten reviewed studies, five [26–29] used Markov modelling techniques to simulate dementia or AD progression. The others included a Monte-Carlo model [30], three decision trees [27, 31, 32] and a regression-based simulation model [33]. There was no evident pattern of model preference in primary, secondary or tertiary prevention studies. In all reviewed studies, little or no rationale was given for the choice of model, and, while model choices did appear appropriate for purpose, it is advisable to include a justification or rationale for the choice of methods [24, 25].

The state-transition Markov models utilised in the five studies were reasonably simple in structure, consisting either of three to four health states (‘non-demented/non-AD’, ‘Mild cognitive impairment’, ‘demented/AD’ and ‘dead’) or five to six states, where ‘demented/AD’ was further broken down into various degrees of severity. While maintaining a simple approach to modelling does require fewer data inputs and, therefore, also exposes the findings to fewer biases, it may also limit the accuracy of the model predictions. It is possible that the choice of health states included is most likely influenced by data availability. Four interventions utilised 12-month cycles [26, 27, 29, 34]; McMahon and colleagues modelled disease progression in 6-week cycles [28]. Cycle length has an impact on model predictions: shorter cycles may improve accuracy, in particular, with regards to survival time, but do require more detailed data.

Two decision tree-based models evaluated screening methods for dementia [31, 35] and one evaluated a preventative treatment [36]. The model by Silverman and colleagues [31] is not transparent, as only a schematic flow of decisions during a screening process is presented, not the actual model structure. Similarly, Dixon et al. only provide a textual description of the model [35]. It is questionable whether the two models could be reconstructed on the basis of the information provided. Tsiachristas and Smith [32] present a stochastic decision model for evaluating the cost-effectiveness of preventative treatment with B-vitamin. The model is not described in great detail, nor represented schematically, although parameters used provide some guidance as to how the model is structured [37].

A parametric Monte Carlo model was built to evaluate the cost-effectiveness of early diagnosis and intervention in AD [30]. The model is described in some detail, and with the aid of specified parameters, it is feasible to presume the model to be reproducible.

McDonnell et al. [33] assessed the cost-effectiveness of a potential treatment compared to standard care by simulating two cohorts of patients with AD using two regression-based simulation models. The models simulated the changes in cognitive decline as measured by Mini-Mental State Examination (MMSE), care setting and mortality of people with AD and compared the costs and effects of the strategies. The models were based on primary data from an epidemiological trial [38] and reproducing the model predictions without access to this data may prove difficult.

#### Comparators used and number of treatments compared

Best practice modelling guidelines recommend that all feasible and practical strategies are considered in decision models [24]. Of the ten studies reviewed, only two assessed more than two comparators. The Monte Carlo model by Weimer and Sager compared two identification strategies (early and delayed), but then applied four hypothetical treatments after diagnosis (pharmacological, nonpharmacological and mix of the two types) [30]. The Markov model constructed by McMahon et al. also compared four screening strategies in base-case, as well as four additional strategies in sensitivity analysis; these included standard screening, a range of different intensities of screening methods, as well as a no-screening option [28].

The remaining four of the five Markov models compared two alternatives – usual care/treatment and new intervention [26, 29, 34]. All three decision tree models compared only two alternatives – no/current intervention with the new intervention [31, 32, 35]. McDonnell and colleagues also compared the impact of two interventions: a hypothetical treatment and standard care [33]. No or little justification for omission of other comparators has been provided by the authors in these studies.

Of three tertiary prevention studies, only one [29] stated explicitly that standard pharmacological intervention was assumed to have been provided alongside the new treatment, in order to compare the new treatment with ‘standard care’ alone. The other two post-diagnostic studies did not explicitly state that they included standard pharmaceutical care alongside their new treatment [33, 34].

#### Time horizon

Decision modelling guidelines recommend that the time horizon of a study should be sufficiently long to capture all the differences of the strategies being assessed; this may require a lifetime horizon [24, 25]. Dementia is a chronic condition for which there is no cure; it also negatively affects survival times [39], suggesting that authors conducting economic evaluations of dementia interventions should pay special attention to ensure all intervention effects are captured.

Only three studies utilised a lifetime time horizon in their model [30, 32, 35] which is the most appropriate approach, in particular for risk-prevention or screening interventions, where health impacts and associated costs may not manifest for a long time after intervention. However, given the late average age of onset for dementia and the age at which patients are exposed to intervention, a 10 or 20 year time horizon may be appropriate; such time horizons were observed in three other included studies [26, 27, 33]. Of the three tertiary prevention interventions reviewed, time horizons were either 10 or 5 years. The latter was utilised by Mirsaeedi-Farahani et al. [34] and Martikainen et al. [29], who adapted a model initially designed for evaluation of pharmaceuticals. However, given that the psychosocial nature of the intervention, the five-year time horizon may not capture all the costs and effects related to the intervention.

### Choice of outcome measure

#### Quality adjusted life years

When using preference-based measures for health outcomes, such as health utilities, to calculate Quality-Adjusted Life Years (QALYs), decision modelling guidelines recommend that the utility weights are incorporated into the model appropriately, and the methods for derivation of utility weights are justified [25].

Four studies employed QALYs as their key outcome measures [26, 28, 32, 34]. McMahon and colleagues [28] classified quality of life measures by disease severity (mild, moderate and severe) as well as residential status (community or nursing home), resulting in six different utilities. The utility weights were obtained from a US study, which used Health Utility Index Mark 2 to gather proxy-rated utilities of 528 caregivers of people with AD [40].

Mirsaeedi-Farahani et al. [34] provided utilities for three levels of AD severity (mild, moderate and severe). These were obtained from eight published studies; no further detail was provided as to how these utility weights were selected or calculated.

Another study calculated QALYs by multiplying years of expected life by utility weights for people with and without dementia, without further breakdown into severity of disease [32]. The utility weights were based on self-completed EQ-5D Visual Analogue Scale (VAS) scores of the general population from the Health Survey for England [32]; it is unclear how these were related to dementia.

Zhang and colleagues [26] also did not distinguish between different stages of disease; QALYs were calculated for people with and without dementia. Utility weights were obtained from a Swedish population study, where general population utilities were gathered using the EQ-5D instrument [41]. It is unclear how the health-related quality of life (HRQoL) of the general population was applied to dementia.

#### Other outcome measures

Three of the ten reviewed studies [27, 30, 33]. included changes in MMSE score [42] as their primary outcome. One study utilised scores collected as part of the study [27] Weimer and Sager [30] used MMSE scores to classify the severity of AD and ascribed varying health care costs to each level of disease severity. MMSE decline with and without treatment was obtained from a range of published literature. McDonnell et al. [33] used MMSE scores to model disease decline; MMSE scores were based on a trial that the study followed [38].

The remaining studies measured outcomes using the number of accurate/additional diagnoses [31, 35] and time to nursing home admission [29].

### Characterisation of disease progression

As both costs and health outcomes are dependent on disease onset and severity, characterisation of disease progression is, arguably, the most critical model input for producing accurate estimates. In many types of decision models, disease progression is directed by progression probabilities, i.e. the probability of shifting from one health state to another. It is advised that transition probabilities should be derived from *“the most representative data sources for the decision problem”* [24].

Four studies based their disease onset and progression parameters on a single study. McDonnell and colleagues [33] used primary data from a large epidemiological trial conducted in the 1990’s to inform disease onset and progression parameters [38]. These were reported by age, gender, residential status and MMSE score. Tsiachristas and Smith [32] based their disease onset and progression parameters on a national report on AD [43]. Zhang et al. [26] synthesised progression probabilities from a single source [44], although it is not transparent how progression probabilities were arrived at. The model by Weimer et al. [30] used disease progression data from a single study of 134 patients on cholinesterase inhibitors [45].

Five studies synthesised this information from two or more studies. McMahon et al. [28] and Martikainen et al. [29] utilised data from US-based studies [40, 46]. Martikainen and colleagues combined data from both papers, but do not provide more detail on how this was done. Another study [34] also utilised data from Neumann et al. [46] and another US-based study [47]. Similarly, Saito et al. also used data on progression of AD from Neumann et al. [46], combining it with another study, reporting on onset of mild cognitive impairment from pre-dementia [48]. Silverman et al. [31] also synthesised onset probabilities from two studies and provided little detail on how this was done.

Finally, Dixon et al. did not specify how disease onset and progression was characterised in their model [35].

### Survival

Survival, or mortality, from dementia was integrated into the reviewed papers in a range of ways. McMahon and colleagues [28] calculated the probability of death from each stage of AD from a Neumann et al. [40], and the probability of death for the non-AD group from US life tables; these were not stratified by age. Weimer and Sager [30] applied a hazard ratio (2.1) to a non-AD population mortality rate to simulate higher rates of mortality. The ratio was obtained from a cardiovascular health study of the US population. Zhang and colleagues [26] populated the model with age-specific mortality for both AD and non-AD groups, obtaining the data from Statistics Sweden; the study assumed the same mortality rate for both groups.

Three papers used the same data sources for mortality/survival as for other disease progression probabilities. Saito et al. obtained mortality data from the same source as the other transition probabilities, by combining two previously published studies [46, 48]. Insufficient details were provided on how these probabilities were combined. Another model [29] also used the same sources for probabilities of death as for other transition probabilities [40, 46]. These were stratified by severity of AD (mild, moderate and severe), but not by age and gender. Mirsaeedi-Farahani et al. [34] also used the same two studies for mortality as for other disease progression probabilities [46, 47]; these were stratified by severity, but not age or gender.

One study [32] used life expectancy data to estimate mortality in their model; UK life tables were used to calculate life expectancy of non-AD group, and AD mortality was based on life expectancy obtained from another UK study [49]. McDonnell et al. calculated probabilities of death based on the results of a study reported in the paper. The probability of death is reported by age, gender, residential status and MMSE score [33].

Finally, two studies did not account for mortality in their models. The study by Silverman et al. [31] did not appear to extend far enough to calculate the impact of screening on mortality, while the other [35] did not include mortality in their parameters, stating that it was ‘beyond the scope of the model’, although a lifetime time horizon was utilised.

### Costs

Decision modelling guidelines recommend that costs included in the model should be consistent with the selected perspective, reflective of all outcomes included in the model and should also be incorporated from data sources in a consistent manner [24, 25]. Overall, costs were consistent with the studies’ perspectives. In terms of consistency of data use, two studies estimated costs from primary data, such as administrative registries and databases [29, 33], three utilised secondary costs [27, 32, 34], and a further four studies used a combination of secondary and primary data for costs, mostly complementing any data missing from primary sources with published information [26, 28, 30, 35]. Silverman and colleagues utilised Medicaid reimbursement rates to estimate their costs [31]. Studies utilising secondary sources included generalised average per patient costs for dementia, of which two used average total annual costs [32, 34] and one included costs per stage of dementia [27]. Studies utilising any primary cost data used unit costs for individual aspects of diagnosis and treatment.

## Discussion

This paper reviews a range of modelling approaches to economic evaluation of NPIs. Although the studies assessed in this paper provide a valuable overview of methodologies available, each comes with limitations. Previous reviews, focusing mainly on pharmacological models, have discussed the limitations in terms of model structure and characterising disease progression [18, 19]. This paper extends this body of knowledge by reviewing decision modelling studies with a special focus on NPIs for dementia.

The majority of tertiary prevention interventions are not designed to replace pharmacological therapy, but rather to complement it. It is reasonable to assume that a NPI, such as cognitive or behavioural interventions, would be used in conjunction with standard pharmacological treatment. However, only one of the three studies modelling tertiary prevention interventions considered the effect of pharmaceuticals. Given that pharmaceuticals do have an effect on cognition [8], omission of this effect poses significant questions over the conclusions of the reviewed studies. Furthermore, only two of the ten reviewed studies examined more than two comparators. A larger number of alternatives courses of action would prove more informative for decision makers.

### Disease progression and outcome measures

Dementia manifests itself in a wide range of ways, including cognitive decline, behavioural and psychological symptoms, as well as deterioration in ability to perform everyday tasks. NPIs for dementia have broad effects, which include improving behavioural and cognitive facets of dementia, as well as improving the overall wellbeing of a patient (and, potentially, their caregiver).

However, this breadth of both impacts of dementia, and interventions to address the disease, was not reflected in the characterisation of disease progression or the breadth of main outcome measures used to measure intervention effectiveness in the reviewed studies. The majority of the interventions utilised measures of cognition, such as MMSE, to model progression of dementia. The use of such measures to model disease progression in NPIs may be problematic, as such interventions often not only focus on slowing progression of the disease, but also on independence of patients and quality of life (both patients’ and caregivers’) such as those assessed in Martikainen [29].

Single-faceted measures of dementia, such as cognition, are also unlikely to fully capture the breadth of the burden imposed by dementia, nor the equally broad impacts of NPIs. This is likely to affect cost and outcome estimates in decision models. An alternative approach could be to characterise disease progression using an alternative concept, such as dependence, or a multi-domain model of disease progression [50, 51]. Broadly, these concepts represent progression of dementia as a function of cognitive, behavioural and activities of daily living aspects of dementia. These allow to classify the burden of disease, and associated costs and outcomes more accurately. It has also been proposed that QALYs and costs associated with each stage of disease can be mapped on to the measure and used in decision modelling [50].

However, the application of these concepts would require additional work and further development of instruments, as neither of the concepts are yet fully developed and ready for application [50–52]. The.

MMSE is a commonly used measure for cognitive ability, it is easy and relatively cheap to administer and can be categorised to define stages of cognitive deterioration. However, MMSE and other measures of cognitive decline are surrogate end-points, and their use in cost-effectiveness analysis has been questioned [53], as it is difficult to establish a cost per incremental change on such a scale. Demographic characteristics of a patient can also be a determinant of cognitive capacity, and this is often not accounted for. Furthermore, measures of cognitive decline do not reflect patients’ or caregivers’ preferences.

A number of reviewed studies used QALYs as an outcome measure. HRQoL measures may capture the effect of an intervention on both morbidity and mortality, and provide a common denominator for comparison of economic evaluations across diseases; however, they also are subject to a number of limitations. For the purposes of brevity, this discussion will focus solely on issues relating to the subject of dementia.

Increase in severity of dementia corresponds with decreased cognitive function, memory loss, and, eventually, physical decline. These changes have a significant effect on the quality of life of a person with the disease, and should be accounted for in an economic evaluation. Two of the four reviewed studies that used QALYs as an outcome measure did not differentiate between stages or severity of dementia, simply providing utility values for either having or not having dementia. This is likely to significantly impact on the accuracy of the findings.

Furthermore, some studies combined utilities from a range of different sources, without providing a justification for why, or an explanation of how. Another important aspect in measuring HRQoL in people with dementia is the loss of cognitive function, which impairs judgement and ability to complete these relatively complex questionnaires. In order to avoid this, proxy-completion by caregivers is often used. However, there is evidence of significant discrepancy between self- and proxy-completed HRQoL measures for people with dementia [54, 55]. It is important to recognise these potential biases in outcome measures for future studies.

### Original vs adapted models

Adapting a model initially designed for evaluation of a pharmacological intervention could be the reason behind the choice of an outcome measure [28, 29]. Such models utilise clinical end-points which are used to measure the effectiveness of a pharmaceutical, which may not be sufficiently broad for NPIs, as previously discussed. Models developed for pharmacological interventions also focus on the period during which medication is taken, often resulting in short time horizons. When applied to a NPI for dementia, this is likely to omit the longer lasting effects of the intervention, and associated costs. In this review, purposefully-designed models tended to have longer time horizons, and also measure outcomes with QALYs, rather than cognitive-based measures.

Decision modelling in NPI for dementia should be conducted using models developed for the purpose. Purpose-developed model are likely to better reflect the features of NPIs, such as broad impacts, more appropriate time-horizons to capture all relevant costs and outcome, and more appropriate characterisation of disease progression.

### Data

A major concern is the availability and accuracy of parameters for disease onset and progression, as well as mortality data. The majority of data on disease onset, progression and mortality used in the included studies were obtained from literature, and, in many cases, from multiple foreign (often US-based) sources.

Synthesising progression probabilities from a range of published sources may be problematic for a number of reasons. First, mortality varies dramatically across the world due to a range of socioeconomic, health care, educational and other factors. Secondly, some of the data were collected in the 1990’s, and both mortality patterns and treatments available have changed significantly since then. This generalisability issue may result in inaccurate predictions of disease-related mortality. However, it is fair to note that this arises due to the lack of appropriate data on disease progression, rather than the quality of research presented. Finally, the methodological differences in calculation of probabilities for disease onset, progression and mortality, and assumptions made during the calculations, may mean that combining data from different studies may reduce the reliability of these inputs.

Data generalisability issues are also highly relevant for mortality data. There is strong evidence to indicate that age, gender, and severity of dementia affect mortality rates [39, 56]. Only one reviewed study parametrised mortality by age, gender, and severity; others focused only on severity. Two studies omitted survival altogether, with one declaring a lifetime horizon for their study. Future decision models of interventions in dementia could consider including mortality and stratifying it by age, gender and severity of dementia for more accurate predictions.

This indicates a need for more detailed and localised data on disease onset, progression and mortality, and stratifying such data by demographic factors such as age and gender. Alternative ways of characterising disease progression, such as dependence and health state utilities, should also be given consideration in future research. Overall, most studies would benefit from employing a more robust approach to economic evaluation in this field, by following a checklist or guidelines for economic evaluation studies such as those outlined by Philips et al. [25] and Caro et al. [24].

### Strengths and limitations

The findings of this review may be limited by the restriction of the review to English-language studies only and only assessing the methodology, and not the findings, of the included studies.

The studies included in this review were relatively heterogenous, representing a range of NPIs as well as modelling methods. However, as we are focusing on methodology, not results, of studies, this should not be a critical factor. We reviewed ten models on NPIs, of which only three evaluated tertiary prevention interventions. This is in stark contrast to the large number of NPIs targeting cognitive, behavioural and functional aspects of dementia [13, 15]. It is difficult to isolate one reason for this disparity; although the issues of data availability could be contributing factors.

This study followed robust methodology outlined by the Cochrane Handbook for Systematic Reviews of Interventions [20]. To the best of our knowledge, this is the first study to review modelling in NPIs for dementia. It is a growing field and the number of new interventions requiring economic evaluation is growing rapidly. Our study provides an insight into methods and data requirements for decision modelling in this area.

## Conclusions

In this paper we reviewed ten economic models that evaluated NPIs for patients with dementia. Economic evaluations of NPIs in dementia should utilise purposefully-developed decision models, and avoid models for evaluation of pharmaceuticals. A major methodological shortcoming identified in this review is the limitation of cognition-focused outcome measures, as they only capture one dimension of a broader range of outcomes NPIs offer. Application of HRQoL measures may also introduce biases and may not completely capture the effects of NPIs. A broader outcome measure, such as dependency, could be considered as an alternative for modelling disease progression. It is also important to account for the effects of pharmacological therapies alongside the NPIs in economic evaluations. We also identified a lack of data availability and accuracy on onset, progression and mortality from dementia. There is a considerable need for development of country or region-specific data based on larger longitudinal studies.

## Additional files

Additional file 1: Search strategies. (DOCX 20 kb)

Additional file 2: Summary of included studies. (DOCX 48 kb)

## References

[1] Wimo A (1998). et al..

[2] Berr C, Wancata J, Ritchie K (2005). Prevalence of dementia in the elderly in Europe. Eur Neuropsychopharmacol.

[3] Ferri CP (2005). Global prevalence of dementia: a Delphi consensus study. Lancet.

[4] Klimova B, Maresova P, Kuca K (2016). Non-pharmacological approaches to the prevention and treatment of Alzheimer's disease with respect to the rising treatment costs. Curr Alzheimer Res.

[5] World Health Organization Dementia: a public health priority. 2012: World Health Organization.

[6] van de Glind, E.M.M., et al., Pharmacological treatment of dementia: a scoping review of systematic reviews. Dement Geriatr Cogn Disord, 2013. 36(3–4): p. 211–228. 10.1159/000353892 23941762

[7] Takeda M (2012). Non-pharmacological intervention for dementia patients. Psychiatry Clin Neurosci.

[8] Tan C-C (2014). Efficacy and safety of donepezil, galantamine, rivastigmine, and memantine for the treatment of Alzheimer's disease: a systematic review and meta-analysis. J Alzheimers Dis.

[9] World Health Organization *First WHO ministerial conference on global action against dementia: meeting report, WHO Headquarters, Geneva, Switzerland, 16–17 March 2015.* 2015.

[10] Olazarán J (2010). Nonpharmacological therapies in Alzheimer’s disease: a systematic review of efficacy. Dement Geriatr Cogn Disord.

[11] Phung KTT, et al. A three-year follow-up on the efficacy of psychosocial interventions for patients with mild dementia and their caregivers: the multicentre, rater-blinded, randomised Danish Alzheimer intervention study (DAISY). BMJ Open. 2013;3(11) 10.1136/bmjopen-2013-003584 PMC3840334 24270834

[12] Brodaty H, Arasaratnam C (2012). Meta-analysis of nonpharmacological interventions for neuropsychiatric symptoms of dementia. Am J Psychiatr.

[13] Cohen-Mansfield J (2005). Nonpharmacological interventions for persons with dementia. Alzheimer's Care Today.

[14] Cohen-Mansfield J (2013). Nonpharmacologic treatment of behavioral disorders in dementia. Curr Treat Options Neurol.

[15] O'Neil, M.E., M. Freeman, and V. Portland, A systematic evidence review of non-pharmacological interventions for behavioral symptoms of dementia. 2011: Department of Veterans Affairs Washington, DC, USA. 21634073

[16] Knapp M, Iemmi V, Romeo R (2013). Dementia care costs and outcomes: a systematic review. International journal of geriatric psychiatry.

[17] Cohen JT, Neumann PJ (2008). Decision analytic models for Alzheimer's disease: state of the art and future directions. Alzheimers Dement.

[18] Green C (2011). Model-based economic evaluation in Alzheimer's disease: a review of the methods available to model Alzheimer's disease progression. Value Health.

[19] Green C (2007). Modelling disease progression in Alzheimer's disease: a review of modelling methods used for cost-effectiveness analysis. PharmacoEconomics.

[20] Higgins J, Green S. Cochrane handbook for systematic reviews of interventions version 5.1. 0. The Cochrane Collaboration. 2013:2011.

[21] Moher D (2009). Preferred reporting items for systematic reviews and meta-analyses: the PRISMA statement. PLoS Med.

[22] Briggs, A.H., K. Claxton, and M.J. Sculpher, Decision modelling for health economic evaluation. 2006: Oxford university press.

[23] Gustavsson A (2017). Current issues and future research priorities for health economic modelling across the full continuum of Alzheimer's disease. Alzheimers Dement.

[24] Caro JJ (2012). Modeling good research practices—overview. Med Decis Mak.

[25] Philips Z (2006). Good practice guidelines for decision-analytic modelling in health technology assessment. PharmacoEconomics.

[26] Zhang Y (2011). Cost-effectiveness of a health intervention program with risk reductions for getting demented: results of a Markov model in a Swedish/Finnish setting. J Alzheimers Dis.

[27] Saito E (2014). Cost effective community based dementia screening: a markov model simulation. Int J Alzheimers Dis.

[28] McMahon PM (2000). Cost-effectiveness of functional imaging tests in the diagnosis of Alzheimer disease. Radiology.

[29] Martikainen J, Valtonen H, Pirttilä T (2004). Potential cost-effectiveness of a family-based program in mild Alzheimer's disease patients. The European journal of health economics: HEPAC : health economics in prevention and care.

[30] Weimer DL, Sager MA (2009). Early identification and treatment of Alzheimer's disease: social and fiscal outcomes. Alzheimers Dement.

[31] Silverman DHS (2002). Evaluating early dementia with and without assessment of regional cerebral metabolism by PET: a comparison of predicted costs and benefits. Journal of nuclear medicine: official publication, Society of Nuclear Medicine.

[32] Knott C (2012). General mental and physical health.

[33] McDonnell J (2001). The cost of treatment of Alzheimer's disease in the Netherlands: a regression-based simulation model. PharmacoEconomics.

[34] Mirsaeedi-Farahani K (2015). Deep brain stimulation for Alzheimer disease: a decision and cost-effectiveness analysis. J Neurol.

[35] Dixon J (2014). Exploring the cost-effectiveness of a one-off screen for dementia (for people aged 75years in England and Wales). International Journal of Geriatric Psychiatry.

[36] Tsiachristas A, Smith AD (2016). B-vitamins are potentially a cost-effective population health strategy to tackle dementia: too good to be true?. Alzheimer's & Dementia: Translational Research & Clinical Interventions.

[37] Smith AD, Refsum H (2016). Homocysteine, B vitamins, and cognitive impairment. Annu Rev Nutr.

[38] Ott A (1995). Prevalence of Alzheimer's disease and vascular dementia: association with education. The Rotterdam study.

[39] Todd S (2013). Survival in dementia and predictors of mortality: a review. International Journal of Geriatric Psychiatry.

[40] Neumann PJ (1999). Cost-effectiveness of donepezil in the treatment of mild or moderate Alzheimer's disease. Neurology.

[41] Burström K, Johannesson M, Diderichsen F (2001). Swedish population health-related quality of life results using the EQ-5D. Qual Life Res.

[42] Folstein MF, Folstein SE, McHugh PR (1975). “Mini-mental state”: a practical method for grading the cognitive state of patients for the clinician. J Psychiatr Res.

[43] Prince M, et al. *Dementia UK: update.* Alzheimer’s. Society. 2014;

[44] Kivipelto M (2006). Risk score for the prediction of dementia risk in 20 years among middle aged people: a longitudinal, population-based study. The Lancet Neurology.

[45] Lopez OL (2005). Alteration of a clinically meaningful outcome in the natural history of Alzheimer's disease by cholinesterase inhibition. J Am Geriatr Soc.

[46] Neumann PJ (2001). Measuring Alzheimer’s disease progression with transition probabilities: estimates from CERAD. Neurology.

[47] Spackman DE (2012). Measuring Alzheimer disease progression with transition probabilities: Estimates from NACC-UDS. Curr Alzheimer Res.

[48] Jack CR, et al. Serial PIB and MRI in normal, mild cognitive impairment and Alzheimer's disease: implications for sequence of pathological events in Alzheimer's disease. Brain. 2009; 10.1093/brain/awp062 PMC2677798 19339253

[49] Lewis F (2014). The trajectory of dementia in the UK—making a difference. OHE consulting.

[50] Green C, Zhang S (2016). Predicting the progression of Alzheimer's disease dementia: a multidomain health policy model. Alzheimers Dement.

[51] McLaughlin T (2010). Dependence as a unifying construct in defining Alzheimer's disease severity. Alzheimers Dement.

[52] McLaughlin T (2010). Assessment of potential measures in models of progression in Alzheimer disease. Neurology.

[53] Johannesson M, Jönsson B, Karlsson G (1996). Outcome measurement in economic evaluation. Health Econ.

[54] Chua K-C (2016). Quality-of-life assessment in dementia: the use of DEMQOL and DEMQOL-proxy total scores. Qual Life Res.

[55] Sheehan BD (2012). Patient and proxy measurement of quality of life among general hospital in-patients with dementia. Aging Ment Health.

[56] Brodaty H, Seeher K, Gibson L (2012). Dementia time to death: a systematic literature review on survival time and years of life lost in people with dementia. Int Psychogeriatr.

[57] Mesterton J (2010). Cross sectional observational study on the societal costs of Alzheimer's disease. Curr Alzheimer Res.

[58] Luengo-Fernandez R, Leal J, Gray A. UK research spend in 2008 and 2012: comparing stroke, cancer, coronary heart disease and dementia. BMJ Open. 2015;5(4) 10.1136/bmjopen-2014-006648 PMC4420948 25869683

[59] Harris GJ (1998). Dynamic susceptibility contrast MR imaging of regional cerebral blood volume in Alzheimer disease: a promising alternative to nuclear medicine. Am J Neuroradiol.

[60] Hux MJ (1998). Relation between severity of Alzheimer's disease and costs of caring. Can Med Assoc J.

[61] Mittelman MS (1996). A family intervention to delay nursing home placement of patients with alzheimer disease: a randomized controlled trial. JAMA.

[62] Fox JP (2001). Estimating the costs of caring for people with Alzheimer disease in California: 2000–2040. J Public Health Policy.

