# Supplementary material for: Decision modelling of non-pharmacological interventions for individuals with dementia: a systematic review of methodologies
Source: Health Econ Rev. 2018 Mar 26;8:8. doi: 10.1186/s13561-018-0192-8 (PMC6755571; doi:10.1186/s13561-018-0192-8)
Supplement: Supplementary file 1 — Search strategies. (DOCX 20 kb) [file 13561_2018_192_MOESM1_ESM.docx]

**Additional file 1:** Search strategies

**PubMed**

| **Search Number** | **Query** | **Results** |
| --- | --- | --- |
| #1 | Search ((alzheimer[Title/Abstract] OR dementia[Title/Abstract])) OR "Alzheimer's disease"[Title/Abstract] | 166,352 |
| #2 | Search (((Cost[Title/Abstract] OR Cost-benefit[Title/Abstract] OR cost-effectiveness[Title/Abstract] OR cost-utility[Title/Abstract])) OR ("cost benefit"[Title/Abstract] OR "cost effectiveness"[Title/Abstract] OR "cost-utility"[Title/Abstract])) OR ("economic evaluation"[Title/Abstract] OR economic[Title/Abstract]) | 471,662 |
| #3 | Search (model[Title/Abstract]) OR "Decision model"[Title] | 1,673.439 |
| #4 | Search ("2000/01/01"[Date - Publication] : "3000"[Date - Publication]) | 14,023,738 |
| #1 AND #2 AND #3 AND #4 | Search ((((((alzheimer[Title/Abstract] OR dementia[Title/Abstract])) OR "Alzheimer's disease"[Title/Abstract])) AND ((((Cost[Title/Abstract] OR Cost-benefit[Title/Abstract] OR cost-effectiveness[Title/Abstract] OR cost-utility[Title/Abstract])) OR ("cost benefit"[Title/Abstract] OR "cost effectiveness"[Title/Abstract] OR "cost-utility"[Title/Abstract])) OR ("economic evaluation"[Title/Abstract] OR economic[Title/Abstract]))) AND ((model[Title/Abstract]) OR "Decision model"[Title/Abstract])) AND ("2000/01/01"[Date - Publication] : "3000"[Date - Publication]) | 370 |

**Scopus**

| **Search Number** | **Query** | **Results** |
| --- | --- | --- |
| #1 | TITLE-ABS-KEY ( alzeimer OR dementia ) AND DOCTYPE ( ar ) AND PUBYEAR > 1999 | 66,448 |
| #2 | ( TITLE-ABS-KEY ( cost  OR  cost-benefit  OR  cost-effectiveness  OR  cost-utility )  OR  TITLE-ABS-KEY ( "Economic Evaluation" )  OR  TITLE-ABS-KEY ( "cost utility"  OR  "cost benefit"  OR  "cost effectiveness" ) )  AND  DOCTYPE ( ar )  AND  PUBYEAR  >  1999 | 875,110 |
| #3 | ( TITLE-ABS-KEY ( model  OR  "Decision Model" ) ) AND  DOCTYPE ( ar  OR  re )  AND  PUBYEAR  >  1999 | 5,383,094 |
| #1 AND #2 AND #3 | ( TITLE-ABS-KEY ( alzeimer  OR  dementia )  AND  DOCTYPE ( ar )  AND  PUBYEAR  >  1999 )  AND  ( ( TITLE-ABS-KEY ( cost  OR  cost-benefit  OR  cost-effectiveness  OR  cost-utility )  OR  TITLE-ABS-KEY ( "Economic Evaluation" ) )  AND  DOCTYPE ( ar )  AND  PUBYEAR  >  1999 )  AND  ( TITLE-ABS-KEY ( model  OR  "Decision Model" )  AND  DOCTYPE ( ar  OR  re )  AND  PUBYEAR  >  1999 ) | 610 |

**Science Direct**

| **Search Number** | **Query** | **Results** |
| --- | --- | --- |
| #1 | pub-date > 1999 and TITLE-ABSTR-KEY(**Alzheimer OR Alzheimer's**) or TITLE-ABSTR-KEY(**Dementia**) | 56,534 |
| #2 | pub-date > 1999 AND (**TITLE-ABSTR-KEY(cost) OR TITLE-ABSTR-KEY(cost-benefit) OR TITLE-ABSTR-KEY(cost-effectiveness) OR TITLE-ABSTR-KEY(cost-utility) OR TITLE-ABSTR-KEY(cost-minimisation) OR TITLE-ABSTR-KEY(cost-minimization) OR TITLE-ABSTR-KEY("Economic Evaluation") OR TITLE-ABSTR-KEY( "cost utility") OR TITLE-ABSTR-KEY("cost benefit") OR TITLE-ABSTR-KEY("cost effectiveness") OR TITLE-ABSTR-KEY("cost minimisation") OR TITLE-ABSTR-KEY("cost minimization")**) | 255,683 |
| #3 | pub-date > 1999 and TITLE-ABSTR-KEY(**Model**) or TITLE-ABSTR-KEY(**"Decision Model"**) | 1,299,188 |
| #1 AND #2 AND #3 | (**pub-date > 1999 and TITLE-ABSTR-KEY(Alzheimer OR Alzheimer's) or TITLE-ABSTR-KEY(Dementia)**) AND (**pub-date > 1999 AND (TITLE-ABSTR-KEY(cost) OR TITLE-ABSTR-KEY(cost-benefit) OR TITLE-ABSTR-KEY(cost-effectiveness) OR TITLE-ABSTR-KEY(cost-utility) OR TITLE-ABSTR-KEY(cost-minimisation) OR TITLE-ABSTR-KEY(cost-minimization) OR TITLE-ABSTR-KEY("Economic Evaluation") OR TITLE-ABSTR-KEY( "cost utility") OR TITLE-ABSTR-KEY("cost benefit") OR TITLE-ABSTR-KEY("cost effectiveness") OR TITLE-ABSTR-KEY("cost minimisation") OR TITLE-ABSTR-KEY("cost minimization"))**) AND (**pub-date > 1999 and TITLE-ABSTR-KEY(Model) or TITLE-ABSTR-KEY("Decision Model")**) | 159 |

**Cochrane**

| **Search Number** | **Query** | **Results** |
| --- | --- | --- |
| #1 | dementia or Alzheimer or "alzheimer's":ti,ab,kw Publication Year from 2000 (Word variations have been searched) | 9,849 |
| #2 | cost or cost-benefit or cost-effectiveness or cost-utility or cost-minimisation or "cost minimisation" or cost-minimization or "cost minimization" or "Economic Evaluation" or "cost utility" or "cost benefit" or "cost effectiveness":ti,ab,kw Publication Year from 2000 (Word variations have been searched) | 65,798 |
| #3 | Model or "decision model":ti,ab,kw Publication Year from 2000 (Word variations have been searched) | 73,527 |
| #4 | #1 and #2 and #3 | 190 |

**NHS EED**

| **Search Number** | **Query** | **Results** |
| --- | --- | --- |
| #1 | (Alzheimer):TI AND (cost OR economic ):TI AND (model) IN DARE, NHSEED, HTA FROM 2000 TO 2017 | 48 |

**Embase**

| **Search Number** | **Query** | **Results** |
| --- | --- | --- |
| #1 | (Alzheimer or Alzheimer's or Dementia).ab. | 496,515 |
| #2 | (Cost or Cost-effectiveness or cost-benefit or cost-utility or cost-minimization or cost-minimisation or (economic or "economic evaluation") or ("cost utility" or "cost benefit" or "cost effectiveness" or "cost minimization" or "cost minimisation")).ab. | 1,260,291 |
| #3 | (model or "decision model").ab. | 4,445,069 |
| #4 | 1 and 2 and 3 | 1355 |
| #5 | limit 4 to yr="2000 -Current" | 1243 |

**EconLit**

| **Search Number** | **Query** | **Results** |
| --- | --- | --- |
| #1 | ab(alzheimer) OR ab((alzheimer's OR dementia))Limits applied | 120 |
| #2 | ab((Cost or Cost-effectiveness or cost-benefit or cost-utility or cost-minimization or cost-minimisation or (economic or "economic evaluation") or ("cost utility" or "cost benefit" or "cost effectiveness" or "cost minimization" or "cost minimisation")))Limits applied | 265,138 |
| #4 | ab(Model) OR ab("decision model")Limits applied | 262,667 |
| #5 | (ab(alzheimer) OR ab((alzheimer's OR dementia))) AND ab((Cost OR Cost-effectiveness OR cost-benefit OR cost-utility OR cost-minimization OR cost-minimisation OR (economic OR "economic evaluation") OR ("cost utility" OR "cost benefit" OR "cost effectiveness" OR "cost minimization" OR "cost minimisation"))) AND (ab(Model) OR ab("decision model")) Additional limits - Date: After 31 December 1999 | 18 |

**PsychInfo**

| **Search Number** | **Query** | **Results** |
| --- | --- | --- |
| #1 | (Abstract:(Model or "Decision Model") AND (Year:[2000 To 9999])) *AND* (Abstract:(Cost or Costeffectiveness or costbenefit or costutility or costminimization or costminimisation or (economic or "economic evaluation") or ("cost utility" or "cost benefit" or "cost effectiveness" or "cost minimization" or "cost minimisation")) AND (Year:[2000 To 9999])) *AND* (Abstract:(Dementia OR Alzheimer OR Alzheimer's) OR Title:(Dementia OR Alzheimer OR Alzheimer's) AND (Year:[2000 To 9999])) | 145 |
