# Supplementary material for: Decision modelling of non-pharmacological interventions for individuals with dementia: a systematic review of methodologies
Source: Health Econ Rev. 2018 Mar 26;8:8. doi: 10.1186/s13561-018-0192-8 (PMC6755571; doi:10.1186/s13561-018-0192-8)
Supplement: Supplementary file 2 — Summary of included studies. (DOCX 48 kb) [file 13561_2018_192_MOESM2_ESM.docx]

Additional file 2: Summary of included studies.

## Preventative and diagnostic interventions

### **McMahon et al.**

McMahon and colleagues [1] compared the cost-effectiveness of functional neuroimaging technique for the diagnosis of Alzheimer’s disease with three other diagnostic strategies using a model with embedded Markov states. The Markov states were taken from a previously published model for economic evaluation of donepezil [2]. The model compared the strategies by running three cohorts through 32,000 trials, where individuals could reside in one of five disease states during each 6-week cycle (‘no AD’, ‘mild AD’, ‘moderate AD’, ‘severe AD’ or ‘dead’). Transition between these states were based on data from CERAD study, as were utility weights for each of these states [2]. The sensitivity of neuroimaging to mild, moderate and severe stages of AD was obtained from a previously published study [3]. Once diagnosed, treatment with donepezil or similar drug was assumed. Costs associated with AD were obtained from published literature [4]. The study was conducted from a societal perspective with an 18-month time horizon. The study concluded that functional neuroimaging was not a cost-effective diagnostic technique.

### **Silverman et al.**

Silverman and colleagues [5] employed decision-tree analysis to investigate the costs and benefits of integrating PET scanning into clinical workup for more accurate diagnosis of AD in a US setting. The decision tree compared two competing strategies: standard practice for expert evaluation of dementia and a new proposed algorithm inclusive of PET scanning. The time horizon of the model is not explicitly stated, but is not longer than one year. Transition probabilities were calculated from a range of published data using ‘standard Bayesian analytic methods’, although little further detail is given. The majority of the costs were defined by Medicare reimbursement values at the time of publication and the rest was obtained from published literature, although it is not clearly stated. Sensitivity and specificity of various diagnostic tests were also found in published literature. The authors do not report conducting sensitivity analyses, although a literature search was conducted to ‘determine reliable means and ranges for all modelled variables’ to ensure no bias was introduced in the model. The findings of this study suggested that introduction of PET scan into clinical workup of AD diagnosis was cost-saving – i.e. less costly and more effective. This was attributed to savings made by providing more timely treatment resulting from more accurate diagnosis due to increased sensitivity and specificity.

### **Weimer and Sager**

Weimer and Sager [6] investigated the costs and benefits of early detection and treatment (using two different interventions – drug therapy for patients and support for caregivers) of patients with AD in a US (Wisconsin) setting. The authors employed a Monte Carlo model due to a large number of variables and the irreversible nature of the condition. The model simulates a cohort of patients with AD through their lifetime, and the annual cognitive decline, and thus provides a distribution of expected net costs and benefits. The progression and staging of AD is defined by MMSE. Cost data were mostly acquired from Wisconsin Department of Health and Family services as well as published data. Probability of cognitive decline and hospitalisation/institutionalisation were derived from Lopez at al. [7] for patients in the study and from Mittelman et al. for the caregiver intervention [8]. Utilities for caregivers and patients were taken from Neumann et al. [2]. This study concluded that early identification of AD may produce positive net social benefits and potentially net savings.

### **Zhang et al.**

Zhang and colleagues [9] investigated the cost-effectiveness of a hypothetical preventative programme for dementia in a Swedish setting. The authors developed a three-state (‘no dementia’, ‘dementia’ and ‘dead’) Markov model with a one year cycle and a 20 year time horizon. The main source of transition probabilities and base line data for the model was CAIDE dementia Risk Score data from a Finnish study [10]. Costs for the hypothetical preventative programme were based on a CVD-prevention programme; other costs and utilities were from previously published (and mostly Sweden-specific) studies. The sensitivity analyses included one way as well as probabilistic sensitivity analyses, which were conducted using Monte Carlo simulations. The reported findings suggest that if the hypothetical programme was run continuously for 20 years, the ICER would be below the willingness to pay level, making the programme potentially cost-effective.

### **Dixon et al.**

Dixon and colleagues [11] built a static decision model to investigate the cost-effectiveness of a one-off screening for dementia among 75 year olds in England and Wales. The structure or the cycle length of the model is not specified. The authors also do not report how progression of dementia was modelled. The benefit from screening was derived from quicker access to social and health care. The care, assumed to be provided to all diagnosed patients, was a combination of medication (acetylcholinesterase inhibitors and memantine), residential care, psychosocial interventions and multi-component carer support. It was assumed that earlier detection prevented additional costs by earlier access to care, which allowed for improved cognition, communication and quality of life. These outcomes were reported either in standardised mean differences (SMD) or in no specific details. Receiving care after diagnosis was also assumed to delay admission to residential care, which was measured in days. One-way sensitivity analyses resulted in a wide range of possible outcomes of introducing a screening programme: from net savings to overall societal costs of over £3,600,000 (using lifetime time-horizon). The study concludes that a one-off screen for 75 year olds may be cost-effective.

### **Saito et al.**

Saito, Nakamoto, Mendez, Mehta and McMurtray [12] constructed a Markov model to evaluate the cost-effectiveness of a community-based screening programme in the US. The screening was assumed to take place at a community Health Care Districts office at an unnamed US location, and participants were recruited through community advertising. Screening involved a 15-minute appointment during which standardised working was recorded and diagnosis of Mild Cognitive Impairment (MCI) and dementia was made through MMSE tests. The model considered six health states (non-demented, 4 stages of dementia or cognitive impairment and death) and simulated the screening programme for 1000 individuals for 10 years, utilising 1 year Markov cycles. Successful detection of all moderate to severe cases was assumed in the model (this was dictated by data collected during screening). Transition probabilities were established from a large national registry, although the methodology applied was unspecified. Surprisingly, the cost data was obtained from a Canadian study on health and ageing. No methodology or results of sensitivity analyses were presented in the paper. The authors reported a total cost saving benefit of 9.8% over a 10 year period compared to no screening and attribute this to decreased time spent in moderate to severe dementia states.

### **Tsiachristas and Smith**

The study [13] investigates the cost-effectiveness of preventing dementia with B-vitamins in the UK using a stochastic decision tree. The model compares providing the B-vitamin supplement (a combination of vitamin B6, vitamin B12, and folic acid) to the population of UK residents aged 60 and over with high levels of tHcy with no treatment. Model structure is not clearly described in the paper. The effectiveness of the intervention is based on a systematic review, which found B-vitamin treatment lower levels of high-plasma total homocysteine (tHcy), a modifiable predictor of cognitive impairment and dementia [14]. The effect size was taken from an observational single-arm study of 93 patients [15]. The model assumes that delay in dementia onset is the product of treatment effectiveness, time to dementia and adherence to treatment. The model utilises life-years and QALYs as outcome measures; these are calculated from UK Life Tables and EQ5D utilities from a general population health survey [16]; utilities differ between ‘no dementia’ and ‘dementia’ states and the ‘dementia’ QALYs are calculated based on the number of people in three stages of severity (mild, moderate and severe). The probability of developing dementia was based on recorded prevalence in the UK [17]. The cost of having dementia was presented as one annual figure based on a UK study of economic burden of dementia [18]; the retail price of the vitamin supplement was used for treatment costs. The authors carried out 10,000 iterations of the model using prespecified distributions for all variables; no further information on distributions of variables was provided. Five univariate sensitivity analyses were carried out. The model found the preventative treatment to be cost-saving.

## Post-diagnostic interventions

### **McDonnell et al.**

McDonnell et al. [19] presented two regression-based simulation models assessing the cost-effectiveness of a potential treatment compared to standard treatment in the Netherlands. The models simulated the changes in cognitive decline as measured by MMSE (Model 1), care setting and death in patients with AD (Model 2). Both models utilised 6-month intervals and follow up until death or a maximum of 10 years. The former was a random-effects linear regression model, which included patient demographics, time in study and interaction between time and study and other variables. The institutional model was a multinomial logistic regression model and estimated the changes in care settings and death. Baseline data came from a longitudinal study (Rotterdam study) spanning 3.4 years. Since only shifts from lower to higher level of care were observed in the study, the institutionalisation model assumed that no changes from higher to lower care were possible. Cost data were not available from the Rotterdam study, so national resources were used instead (Dutch Ministry of National Health, Welfare and Sport, Central Bureau for Statistics and published yearbooks on costs of care services). No assumptions were made about the nature or cost of the hypothetical treatment. The study reported that 10 year per patient costs remained under 100,000 euro for all scenarios, and slowing cognitive impairment resulted in longer life expectancy and less time spent in nursing homes.

### **Martikainen et al.**

Martikainen and colleagues [20] conducted an economic evaluation of a cognitive-behavioural family intervention for AD in a Finnish setting. The authors adapted a Monte Carlo Markov model from Neumann et al. [2] which was originally designed for economic evaluation of donepezil, to a Finnish context. The model consisted of 4 health states, had annual Markov cycles and a 5-year horizon. The model used cognitive function to characterise progression and assumed caregivers’ health related quality of life was dependant on AD patients’ disease progression. Cost data were collected from a national report and primary sources. Treatment effectiveness data were taken from a previously published study [8] and utility values were adapted from the original model [2]. The authors found the intervention to provide greater net benefits than current practice, which was a combination of community services and periods of institutionalisation.

### **Misraeedi-Farahani et al.**

The study utilised a purpose-built Markov model to investigate the required effectiveness of Deep Brain Stimulation (DBS) therapy in order to be considered cost-effective in patients with AD. DBS was assumed to slow down progression of AD. The disease progression was modelled using data from CERAD. Direct and indirect costs were considered, and obtained from published literature [21]. The model consisted of five health states (Minimal, Mild, Moderate and Severe AD, as well as death) and estimated the costs (direct and indirect) and effects over 5 years. The hypothetical effect of DBS was considered to be return to the ‘Minimal’ state; as the effectiveness of DBS on intervention was unknown, the authors ranged it from zero to 100%. The study found that DBS 80% effective in order to be more effective and less costly than standard treatment. The results were reported as total costs and total effects after 5 years (discounted at 3%), although no incremental analysis was performed. One-way sensitivity analyses did not yield major variation in cost/QALY.

### **References:**

1. McMahon, P.M., et al., *Cost-effectiveness of Functional Imaging Tests in the Diagnosis of Alzheimer Disease.* Radiology, 2000. **217**(1): p. 58-68.

2. Neumann, P.J., et al., *Cost-effectiveness of donepezil in the treatment of mild or moderate Alzheimer's disease.* Neurology, 1999. **52**(6): p. 1138-1145.

3. Harris, G.J., et al., *Dynamic susceptibility contrast MR imaging of regional cerebral blood volume in Alzheimer disease: a promising alternative to nuclear medicine.* American Journal of Neuroradiology, 1998. **19**(9): p. 1727-1732.

4. Leon, J., C.K. Cheng, and P.J. Neumann, *Alzheimer's disease care: costs and potential savings.* Health Affairs, 1998. **17**(6): p. 206-216.

5. Silverman, D.H.S., et al., *Evaluating early dementia with and without assessment of regional cerebral metabolism by PET: a comparison of predicted costs and benefits.* Journal of nuclear medicine : official publication, Society of Nuclear Medicine, 2002. **43**(2): p. 253-266.

6. Weimer, D.L. and M.A. Sager, *Early identification and treatment of Alzheimer's disease: Social and fiscal outcomes.* Alzheimer's & Dementia, 2009. **5**(3): p. 215-226.

7. Lopez, O.L., et al., *Alteration of a Clinically Meaningful Outcome in the Natural History of Alzheimer's Disease by Cholinesterase Inhibition.* Journal of the American Geriatrics Society, 2005. **53**(1): p. 83-87.

8. Mittelman, M.S., et al., *A family intervention to delay nursing home placement of patients with alzheimer disease: A randomized controlled trial.* JAMA, 1996. **276**(21): p. 1725-1731.

9. Zhang, Y., et al., *Cost-Effectiveness of a Health Intervention Program with Risk Reductions for Getting Demented: Results of a Markov Model in a Swedish/Finnish Setting.* Journal of Alzheimer's Disease, 2011. **26**(4): p. 735-744.

10. Kivipelto, M., et al., *Risk score for the prediction of dementia risk in 20 years among middle aged people: a longitudinal, population-based study.* The Lancet Neurology, 2006. **5**(9): p. 735-741.

11. Dixon, J., et al., *Exploring the cost-effectiveness of a one-off screen for dementia (for people aged 75years in England and Wales).* International Journal of Geriatric Psychiatry, 2014. **30**(5): p. 446-452.

12. Saito, E., et al., *Cost effective community based dementia screening: a markov model simulation.* International journal of Alzheimer's disease, 2014. **2014**: p. 103138.

13. Tsiachristas, A. and A.D. Smith, *B-vitamins are potentially a cost-effective population health strategy to tackle dementia: Too good to be true?* Alzheimer's & Dementia: Translational Research & Clinical Interventions, 2016. **2**(3): p. 156-161.

14. Smith, A.D. and H. Refsum, *Homocysteine, B Vitamins, and Cognitive Impairment.* Annual Review of Nutrition, 2016. **36**(1): p. 211-239.

15. Oulhaj, A., et al., *Homocysteine as a predictor of cognitive decline in Alzheimer's disease.* International Journal of Geriatric Psychiatry, 2010. **25**(1): p. 82-90.

16. Knott, C., *General mental and physical health.* Health Survey for England, 2012.

17. Lewis, F., et al., *The trajectory of dementia in the UK—Making a difference.* OHE Consulting, 2014.

18. Luengo-Fernandez, R., J. Leal, and A. Gray, *UK research spend in 2008 and 2012: comparing stroke, cancer, coronary heart disease and dementia.* BMJ Open, 2015. **5**(4).

19. McDonnell, J., et al., *The cost of treatment of Alzheimer's disease in The Netherlands: a regression-based simulation model.* PharmacoEconomics, 2001. **19**(4): p. 379-390.

20. Martikainen, J., H. Valtonen, and T. Pirttilä, *Potential cost-effectiveness of a family-based program in mild Alzheimer's disease patients.* The European journal of health economics : HEPAC : health economics in prevention and care, 2004. **5**(2): p. 136-142.

21. Fox, J.P., et al., *Estimating the Costs of Caring for People with Alzheimer Disease in California: 2000–2040.* Journal of Public Health Policy, 2001. **22**(1): p. 88-97.
